# Targeting Psychological Pain After a Suicide Attempt: Scoping Review and Intervention Protocol

**DOI:** 10.3390/jcm15083124

**Published:** 2026-04-20

**Authors:** Laura Comendador, Diego J. Palao, Antoni Sanz, Jorge Andreo-Jover, Enrique Baca-García, Maria Luisa Barrigón, María Teresa Bobes-Bascarán, María Ángeles Botí, Marina Diaz-Marsá, Matilde Elices, Ariel Gaona-Casas, Ana González-Pinto, Iria Grande, Luis Jiménez-Treviño, Ángela Palao-Tarrero, Anna Pedrola-Pons, Natalia Roberto, Pilar Alejandra Saiz, Elizabeth Suarez-Soto, Alejandro de la Torre-Luque, The SURVIVE Consortium, Adrián Alacreu-Crespo, Ana Isabel Cebrià, Victor Perez-Sola

**Affiliations:** 1 Department of Psychiatry and Forensic Medicine, Faculty of Medicine, Universitat Autònoma de Barcelona, 08193 Cerdanyola del Vallès, Spain; laura.comendador@autonoma.cat (L.C.);; 2 Department of Mental Health, Parc Taulí University Hospital, Institut d’Investigació i Innovació Parc Taulí (I3PT-CERCA), Unitat de Neurociència Translacional Parc Taulí-I3PT-INc-UAB, Plaça Torre de l’Aigua, s/n, 08208 Sabadell, Spain; 3 Centro de Investigación Biomédica en Red de Salud Mental (CIBERSAM), Instituto de Salud Carlos III, 28029 Madrid, Spainluisjimeneztre@gmail.com (L.J.-T.);; 4 Department of Basic, Developmental, and Educational Psychology, Faculty of Psychology, Universitat Autònoma de Barcelona, 08193 Cerdanyola del Vallès, Spain; 5 Hospital La Paz Institute for Health Research (IdiPAZ), 28046 Madrid, Spain; 6 Department of Psychiatry, Instituto de Investigación Sanitaria de la Fundación Jiménez Díaz (IIS-FJD), 28040 Madrid, Spain; 7 Department of Psychiatry, Hospital Universitario Fundación Jiménez Díaz, 28040 Madrid, Spain; 8 Department of Psychiatry, Hospital Universitario General de Villalba, 28400 Madrid, Spain; 9 Department of Psychiatry, Hospital Universitario Rey Juan Carlos, 28933 Madrid, Spain; 10 Department of Psychiatry, Hospital Universitario Infanta Elena, 28342 Madrid, Spain; 11 Department of Psychiatry, Universidad Autónoma de Madrid (UAM), 28049 Madrid, Spain; 12 Institute of Psychiatry and Mental Health, Hospital General Universitario Gregorio Marañón, IiSGM School of Medicine, Complutense University, 28009 Madrid, Spain; 13 Department of Psychology, Universidad de Oviedo, 33003 Oviedo, Spain; 14 Instituto de Investigación Sanitaria del Principado de Asturias (ISPA), 33011 Oviedo, Spain; 15 Instituto Universitario de Neurociencias del Principado de Asturias (INEUROPA), 33011 Oviedo, Spain; 16 Servicio de Salud del Principado de Asturias (SESPA), 33001 Oviedo, Spain; 17 Bipolar and Depressive Disorders Unit, Hospital Clinic de Barcelona, 08036 Barcelona, Spain; 18 Institut d’Investigacions Biomèdiques August Pi i Sunyer (IDIBAPS), 08036 Barcelona, Spain; 19 Hospital Clinico San Carlos, 28040 Madrid, Spain; 20 Department of Legal Medicine, Psychiatry and Pathology, Faculty of Medicine, Universidad Complutense de Madrid, 28040 Madrid, Spain; 21 Neuroscience Research Program, Hospital del Mar Research Institute, Hospital del Mar, 08003 Barcelona, Spain; 22 BIORABA, Department of Psychiatry, Hospital Universitario de Alava, 01009 Vitoria, Spain; 23 Department of Psychiatry, Universidad del País Vasco/Euskal Herriko Unibertsitatea (UPV/EHU), 01006 Vitoria, Spain; 24 Departament de Medicina, Facultat de Medicina i Ciències de la Salut, Universitat de Barcelona (UB), 08036 Barcelona, Spain; 25 Institute of Neurosciences (UBNeuro), 08035 Barcelona, Spain; 26 Department of Psychiatry, Universidad de Oviedo, 33006 Oviedo, Spain; 27 Department of Psychiatry, Clinical Psychology and Mental Health, La Paz University Hospital, 28046 Madrid, Spain; 28 Faculty of Health Sciences, International University of Valencia (VIU), 46002 Valencia, Spain; 29 Departamento de Psicología y Sociología, Universidad de Zaragoza, 50009 Teruel, Spain; 30 Department of Clinical and Health Psychology, Faculty of Psychology, Universitat Autònoma de Barcelona, 08193 Cerdanyola del Vallès, Spain; 31 Mental Health Institute, Hospital del Mar, 08003 Barcelona, Spain; 32 Department of Experimental and Health Sciences, Pompeu Fabra University, 08002 Barcelona, Spain

**Keywords:** suicide, suicidal behaviour, psychache, psychological pain, therapeutic interventions, clinical outcomes

## Abstract

**Background/Objectives**: Psychological pain—also termed psychache or mental pain—has been suggested to constitute a relevant factor in the emergence of suicidal behaviour. Despite conceptual advances, empirical research on interventions specifically designed to alleviate psychological pain in individuals who have attempted suicide remains scarce. The present scoping review maps existing psychological and pharmacological interventions targeting psychological pain, identifies their core components, delineates gaps for future research, and proposes a therapeutic intervention protocol. **Methods**: Literature was searched through PubMed, PsycInfo, and ClinicalTrials.gov (until October 2025) using combinations of the terms suicide, psychache, psychological pain, intervention, treatment, therapy, pharmacological treatment, and psychotherapy. Both randomised controlled trials, non-randomised controlled trials, and literature reviews were included. **Results**: Evidence indicates that few interventions explicitly target psychological pain. Most suicide-specific therapies indirectly address components of psychological pain—such as unbearable affect, loss of meaning, and social disconnection. Narrative-based, emotion regulation, and acceptance-based therapies appear promising. Emerging pharmacological approaches may relieve psychological pain; however, further evidence is required. **Conclusions**: Integrating psychological pain as a therapeutic focus—through narrative, tolerance-building, and relational strategies—may enhance post-attempt interventions. Future trials should systematically measure psychological pain and test its role as a mediator of suicidal outcomes.

## 1. Introduction

In recent years, psychological pain has been increasingly recognised as a core construct underlying a wide range of psychiatric conditions [1], particularly those associated with emotional distress, impaired self-concept, and suicidal behaviour. The interest in psychological pain in mental health research and intervention is growing due to its transdiagnostic characteristics [1,2], adding to its potential role as a proximal mechanism linking subjective suffering to adverse clinical outcomes, including suicidal ideation and behaviour [3].

Psychological pain—also termed ‘psychache’, ‘mental pain’, or ‘emotional pain’—is defined as an internal disturbance encompassing beliefs, thoughts, emotions, and behaviours intrinsic to the experience of suffering [4]. The conceptualisation of psychological pain is difficult due to the great quantity of terminology defining the construct [5,6]. Although the terminology varies across theoretical frameworks, recent qualitative research definitions describe psychological pain as an unpleasant, immediate, identity-disrupting, and invisible experience [7]. Additionally, Meerwick and Weiss [7] made an effort to integrate the different definitions of psychological pain, resulting in the definition of several key characteristics, including unpleasant feelings, disintegration of self, permanence, and unsustainability [5,6], as well as subjectivity in the way this pain is felt [8]. Moreover, the emergence of psychological pain can be conceptualised as a signal of a threatened sense of self in response to bodily, psychological, existential, or social adversity [7]. Therefore, psychological pain is a fundamental concept in one’s definition of self, meaning that intense psychological pain could call into question a person’s personal worth.

These characteristics of psychological pain may be the cause of its direct relationship with suicidal ideation and behaviours. Shneidman [8,9] conceptualised psychological pain as an intolerable and perturbing mental state characterised by overwhelming negative emotions—such as guilt, loneliness, and fear. From this perspective, suicide represents an attempt to cease awareness of unbearable psychological pain, a notion encapsulated in Shneidman’s axiom, ‘without psychache, there is no suicide’ [10]. This axiom subsequently influenced contemporary theoretical models, including the Three-Step Theory [11] where suicidal ideation appears when psychological pain and hopelessness are present together.

Experiencing intolerable levels of psychological pain has been associated with increased suicide risk [12] in both clinical and non-clinical populations [13]. Psychological pain is involved in all stages of suicidology; a previous meta-analysis showed that higher levels of psychological pain are associated with higher odds of current or lifetime suicidal ideation or suicide attempt independently of depression [14]. Indeed, patients with high psychological pain at inclusion have more possibilities to have a suicidal event (suicide, suicide attempt, or hospitalisation for suicide) 1 year later [15]. Given its relevance to suicide, psychological pain has become an important clinical outcome for intervention, as well as a therapeutic target in suicide prevention [1].

### 1.1. Conceptual Insights on Psychological Pain as a Bridge to Suicidal Tendencies

Several research links the conceptual background of psychological pain with suicidality. Berardelli et al. [16] demonstrated that interpersonal needs, psychological pain, and hopelessness are interlinked among psychiatric inpatients with suicidal ideation, with psychological pain mediating the relationship between perceived burdensomeness and suicidality. Similarly, Ballard et al. [17] found that psychological pain and hopelessness prospectively predicted suicidal thoughts, underscoring the dynamic interplay between emotional suffering and cognitive despair, as specified in the Three-Step Theory of Suicide. Adding further support to the central role of psychological pain, Campos et al. [18] showed that psychological pain mediated the association between the frequency of psychological symptoms and suicidal ideation, and partially mediated the relationship between symptom intensity and suicidal ideation in a community sample. Expanding on this evidence with a more detailed analytical approach, Li et al. [19] applied network analysis to unravel the dimensional links between psychological distress, meaning in life, and suicidal ideation. Their findings revealed that psychological distress was strongly related to hopelessness and sleep-related disorders, suggesting pathways through which unbearable emotional distress can escalate to suicidal ideation. Conversely, the presence of meaning in life emerged as a key protective bridge node, showing negative associations with psychological pain, hopelessness, and pessimism, and highlighting meaning-seeking processes as central buffers against suicide risk. Overall, these findings reinforce Shneidman’s [20] formulation positioning psychological pain as a core mechanism in suicidality, suggesting that psychological pain not only co-occurs with distress but also actively explains how general psychological suffering translates into suicidal thinking.

Regarding suicide behaviours, in depressed patients with suicide attempts, Alacreu-Crespo et al. [21] demonstrate that melancholic symptoms of depression (i.e., sadness, guilt, lack of initiative, and loss of appetite) and physical pain are the variables with greater predictive value of the psychological pain construct. Pompili et al. [22,23] in a cohort of psychiatric patients, demonstrated the link of psychological pain with suicide attempt and suicide intent, showing that patients with more severe psychiatric disorders—specially depression—are more vulnerable to suicide behaviour when psychological pain appeared. Moreover, they found that psychological pain mediates the relationship between childhood trauma and suicide attempt [22]. Finally, Calati et al. [24] using a network perspective, showed that the most proximal nodes to suicide attempts are psychological pain and acquired capability for suicide. Additionally, higher levels of psychological pain have been reported more frequently among younger populations [21,23], women, and those who are divorced, widowed, or unemployed [23].

Especially interesting is the connection of psychological pain with cognition and brain functioning in the prediction of suicide attempt or ideation. Cáceda et al. [25] showed a connection between choice impulsivity and high psychological pain with suicide attempts and ideation. Similarly, Alacreu-Crespo et al. [26] demonstrated that people with high psychological pain who perform better decision-making are less likely to attempt suicide. Additionally, Jollant et al. [27] found that psychological pain was significantly correlated with inhibitory control, as well as with other cognitive measures such as verbal fluency and decision-making, suggesting that core cognitive impairments linked to suicide risk are closely intertwined with the experience of psychological pain. Brain activity studies during social exclusion procedures point to the insula, anterior cingulate cortex [28], and the orbitofrontal cortex [29] as the brain areas linking suicidality with psychological pain. Therefore, these results suggest that the psychological pain experienced by suicidal patients has implications in areas of the brain related to emotional interpretation and pain sensations (insula and cingulate), as well as in areas involved in executive control. All of these previous studies show the implications of psychological pain on the processes involved in the transition from ideation to action.

### 1.2. Intervention on Psychological Pain

From a clinical perspective, several therapeutic approaches—such as Cognitive Behavioural Therapy (CBT), Dialectical Behaviour Therapy (DBT), Acceptance and Commitment Therapy (ACT), and other interventions focused on emotional regulation and cognitive restructuring—have shown effectiveness in reducing suicidal ideation [30,31,32]. Despite the importance of psychological pain in suicide, many established psychotherapies address psychological pain only indirectly, and there is a scarcity of interventions explicitly targeting psychological pain as a primary outcome [33]. Overall, the available evidence for psychological pain remains limited and fragmented, making it difficult to consolidate specific and standardised protocols for addressing psychological pain.

In the pharmacological field, psychological pain is also neglected; treatment guidelines focus on the importance of optimising treatment of the psychiatric conditions known to be associated with suicide risk. It is recommended to use an enhanced medication management strategy that includes maintaining a supportive, non-judgemental therapeutic relationship; flexibility; collaboration; consideration of combining medications with evidence-based non-pharmacological strategies; and ongoing safety planning [34].

Despite this evidence and its potential utility for clinical risk detection and its role as a potential therapeutic target, routine assessment of psychological pain has not yet been integrated into standard clinical practice [35,36]. The present scoping review synthesises empirical findings on interventions—psychological and pharmacological—designed to alleviate psychological pain among individuals experiencing suicidality or post-attempt crises. The objectives are: (1) to analyse the most effective intervention components for treating psychological pain; (2) to explore psychological and pharmacological approaches, highlighting their differences, strengths, and limitations; and (3) to draft a therapeutic protocol evidence-based that takes into account psychological pain.

## 2. Materials and Methods

The review was conducted following the recommendations of the PRISMA Extension for Scoping Reviews (PRISMA-ScR; Preferred Reporting Items for Systematic reviews and Meta-Analyses extension for Scoping Reviews) [37] (Supplementary File S1). Although a formal protocol was not pre-registered, the review process followed a structured and predefined strategy regarding search, eligibility criteria, and data extraction.

A literature search was conducted using PubMed and PsycInfo databases. ClinicalTrials.gov was consulted for ongoing or unpublished trials. No additional citation tracking procedures were performed. Reference lists of included studies and citing references were not systematically examined, and no citation index searches or automated alerts were used. The search included English- and Spanish-language articles published until October 2025 without restrictions on publication year and used combinations of the following MeSH keywords: suicide; psychache; psychological pain; mental pain; social pain; emotional pain; emotional suffering; psychic pain; intervention; treatment; therapy; pharmacological treatment; pharmacotherapy; psychotherapy. The drafted electronic search strategy for each database is included in the Supplementary Material (Supplementary File S2). Inclusion criteria comprised studies addressing adult populations (≥18 years) with suicidal behaviour and psychological pain in psychiatric or emergency settings. Studies including participants of both sexes were considered eligible, and no restrictions regarding sex or gender were applied during the selection process. Eligible evidence included randomised controlled trials, non-randomised controlled trials, and literature reviews. Exclusion criteria were studies with pediatric-only populations and non-English or Spanish publications. The search strategy was specifically developed for the purposes of the present review based on the study objectives and predefined eligibility criteria. Search strategies from previous literature reviews were not adapted or reused.

Titles and abstracts were screened independently by two reviewers (L.C. and A.I.C.). Full-text articles were retrieved for records deemed potentially relevant. Final inclusion decisions were reached by consensus, with disagreements adjudicated by a third reviewer (A.A.). Data extraction was performed based on the study design, sample characteristics, setting, definitions and measures of psychological pain, intervention characteristics (when applicable), and main outcomes by two reviewers (L.C. and A.I.C.). The duplicate articles were manually removed by the first reviewer (L.C.). As the available literature on psychological pain and suicidal behaviour interventions is heterogeneous in terms of designs, populations and outcomes—ranging from observational studies to clinical trials—it was not possible to conduct a quantitative synthesis. Therefore, a narrative synthesis was chosen as an appropriate methodological approach to comprehensively integrate existing evidence, identify common patterns and highlight relevant knowledge gaps.

The literature was selected for inclusion based on its relevance to the following areas: conceptualisation of suicide attempts and psychological pain in different populations, clinical manifestations and correlates associated with psychological pain, diagnostic frameworks used for its assessment, available psychological and pharmacological interventions, and strategies aimed at suicide prevention and the treatment of psychological pain. A formal critical appraisal of the included sources was not conducted. This is consistent with the objectives of scoping reviews, which aim to map the available evidence. Given the heterogeneity of study designs, the focus was placed on providing a descriptive synthesis rather than a quality-based evaluation.

It should be noted that, in addition to synthesising the available evidence, the present methodological approach was designed to combine a structured narrative review with the development of an operational therapeutic protocol. Specifically, the identification of core therapeutic components derived from the literature informed the subsequent design of a manualised intervention aimed at directly targeting psychological pain in individuals after a suicide attempt. This integrative framework was intended to ensure that the proposed protocol was grounded in existing empirical evidence while addressing limitations of current models.

## 3. Results

**Figure 1:**
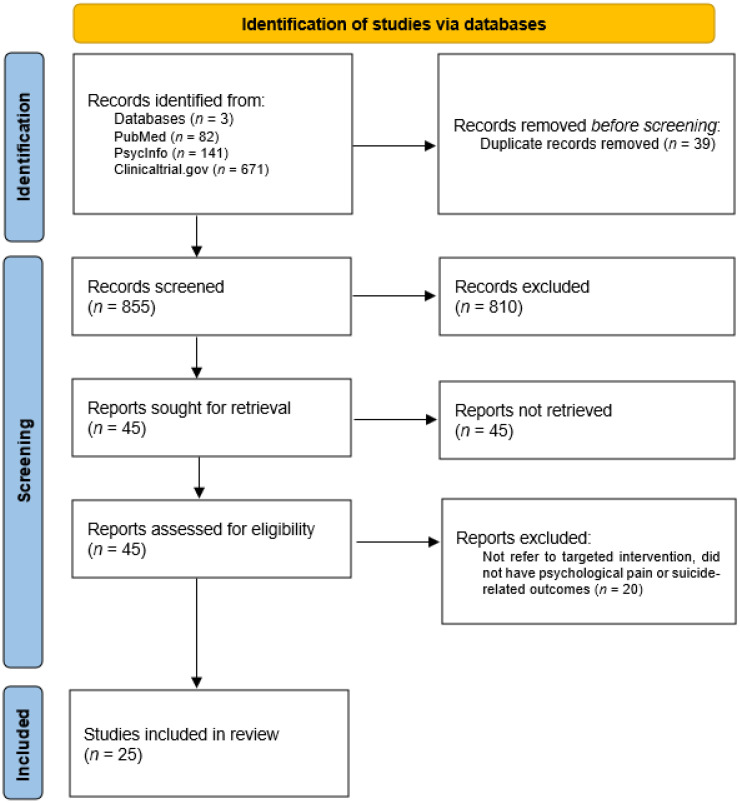
PRISMA flow diagram.

The initial search revealed a total of 855 candidate publications, from which 45 articles were retained for full-text review. Finally, 25 studies were included in the review. We excluded studies that did not refer to targeted intervention or did not have psychological pain or suicide-related outcomes. The article selection process was described in a PRISMA flowchart (Figure 1).

The main characteristics of the included studies are summarised in Table 1. Given the heterogeneity of the included studies—comprising randomised controlled trials (RCT), non-randomised designs, and reviews—the following synthesis aims to provide a descriptive overview of the current evidence on interventions related to psychological pain in suicidal populations. The focus is placed on identifying common therapeutic approaches, reported effects, and methodological limitations.

### 3.1. Psychological Interventions Targeting Psychological Pain

#### 3.1.1. Third-Generation Therapies: Acceptance and Commitment Therapy (ACT) and Mindfulness-Based Cognitive Therapy (MBCT)

Evidence from controlled studies has examined the potential effects of third-generation psychological interventions on psychological pain and suicide-related outcomes. Ducasse et al. [38] conducted a RCT comparing ACT with relaxation training among patients with suicidal behaviours. ACT was associated with reductions in psychological pain (ACT β [SE] = −0.54 [0.09]; relaxation β [SE] = −0.04 [0.09]; *p* = 0.001) at post-treatment, as well as in other suicide-related outcomes. ACT’s focus on experiential acceptance and values-driven behaviours has been proposed as potentially relevant for modifying the relationship individuals have with their internal experiences. Subsequent studies by Ducasse et al. [39] showed that mindfulness-based gratitude diaries significantly decreased, immediately but transiently, psychological pain (t = 6.65, *p* < 0.001) and suicidal ideation (t = 4.34, *p* < 0.001) among inpatients. Moreover, Bindu & Vargas [40] developed and tested a mindfulness-based cognitive restructuring (MBCT) programme among adolescents. Using a randomised controlled design, they reported that the intervention was associated with reductions in psychological distress, hopelessness, and suicidal ideation compared to a control group (*p* < 0.001). The effect size was large, and improvements in the dimensions of emotional pain and cognitive hopelessness suggested that combining mindfulness skills with cognitive restructuring may help young people reframe distressing thoughts, tolerate internal discomfort, and foster future-oriented thinking.

#### 3.1.2. Cognitive Behavioural Therapy for Suicide Prevention (CBT-SP)

Several studies have described cognitive-behavioural approaches for suicide prevention, including interventions not explicitly designed to target psychological pain. Bryan [45] described CBT-SP as a structured intervention incorporating skills training, cognitive restructuring, and relapse prevention. Although not designed exclusively for psychological pain, CBT-SP may indirectly address it through increased distress tolerance and cognitive flexibility. Case-based studies [44] suggest CBT imagery interventions targeting suicidality may contribute to reframing the emotional meaning of psychological pain, fostering greater self-efficacy.

#### 3.1.3. Interpersonal and Relational Approaches

Interpersonal and relational models have also been described in the context of suicide prevention and the management of emotional distress. The PROTECT framework [46] introduced a relational-safety-based model for suicide prevention emphasising interpersonal connection and empathic communication. Chammas et al. [47] evaluated suicide prevention measures in inpatient psychiatric settings and reported that strengthening relational engagement and monitoring emotional pain was associated with improved safety outcomes. These findings are consistent with theoretical models such as the Interpersonal Theory of Suicide [48], which propose that perceived burdensomeness and thwarted belongingness are associated with emotional pain.

#### 3.1.4. Brief Contact and Crisis-Focused Interventions

Brief contact and crisis-focused interventions have been evaluated in individuals following suicide attempts, focusing on immediate support and crisis management. O’Connor et al. [41] and Stanley & Brown [49] tested brief psychological-based interventions for individuals following suicide attempts. These interventions aimed to provide structured emotional support, crisis planning, and connection reinforcement. While reductions in reattempts were modest, qualitative analyses suggest that empathic contact may reduce ‘emotional loneliness’ and acute distress—core features of psychological pain. Also, crisis hotline studies [42] reported immediate reductions in psychological pain (F = 181.4, *p* < 0.001), suicidal ideation (F = 130.8, *p* < 0.001), and hopelessness (F = 112.8, *p* < 0.001).

#### 3.1.5. Psychological Pain Theory-Based Cognitive Therapy

Zou et al. [43] piloted Psychological Pain Theory-Based Cognitive Therapy in patients with major depressive disorder and suicidal ideation. The intervention, delivered over eight weeks, was associated with reductions in psychological pain (F = 3.36, *p* < 0.05, ηp^2^ = 0.20), depression (F = 3.71, *p* < 0.05, ηp^2^ = 0.21), and suicidal ideation (F = 5.08, *p* < 0.05, ηp^2^ = 0.27). Notably, patients receiving the intervention showed maintenance of low suicidal ideation at four-week follow-up, whereas control participants receiving usual psychological care showed an increase in suicidal thoughts.

### 3.2. Ongoing or Unpublished Trials on Psychological Interventions for Suicide Prevention

Several ongoing or recently completed trials aim to investigate interventions targeting suicidal ideation, suicidal behaviours, and associated mental distress, although results are not yet published. The ‘Pain Perception in Suicidal Vulnerability’ study (NCT02915679) investigates physical and social pain sensitivity in depressed individuals with and without a history of suicide attempts. Using social exclusion task (Cyberball), the study aims to identify potential trait-level vulnerabilities related to suicidal behaviour. The ‘ASSIP vs. ACT’ trial (NCT07132099) compares the Attempted Suicide Short Intervention Program (ASSIP) with ACT and Treatment As Usual (TAU) in adults with recent suicide attempts. Primary outcomes include suicidal ideation and psychological pain, measured pre- and post-intervention and at one-month follow-up, providing comparative evidence on brief psychotherapeutic approaches. Finally, the ‘Group CBT for Psychological Sub-health’ trial (NCT05913349) evaluates a brief, structured CBT group intervention for individuals experiencing psychological distress. The study incorporates wearable devices and mobile apps to collect behavioural and physiological data, aiming to explore mechanisms of treatment response and scalability of digital-supported interventions. Together, these studies highlight growing interest in mechanisms of psychological pain and scalable intervention models.

### 3.3. Pharmacological Approaches

Pharmacological treatments specifically targeting psychological pain remain limited. Nobile et al. [51] explored the role of opioids in social pain and suicidality, finding preliminary evidence that low-dose buprenorphine may attenuate affective distress by modulating the endogenous opioid system. Conejero et al. [52] noted that ketamine’s rapid reduction in suicidal ideation might partially derive from its impact on psychological pain. Complementing this, Ballard et al. [50] examined clinical indicators of the suicide crisis and the response to ketamine in a cohort of high-risk individuals. Their findings indicated that suicidal ideation, depression, hopelessness, and psychological pain were heightened during acute suicidal states and decreased after ketamine infusion, suggesting ketamine’s potential utility in rapidly alleviating core emotional drivers of suicidal crises. Importantly, traumatic stress symptoms also improved, underscoring the multifactorial nature of acute suicidality and highlighting the value of targeting psychological pain alongside traditional mood symptoms. These findings suggest potential short-term benefits, although long-term sustainability remains uncertain.

### 3.4. Literature Reviews of Interventions in Psychological Pain

Evidence syntheses have consistently described psychological pain as a clinically relevant construct. Courtet & Saiz [54], in ‘Let’s Move Towards Precision Suicidology’, proposed personalised interventions integrating psychometric, biological, and experiential markers, including measures of psychological pain, to tailor suicide prevention strategies. D’Anci et al. [53] systematically reviewed treatments for suicide prevention, reporting that cognitive-behavioural and mindfulness-based interventions were among the most frequently supported approaches. Holm et al. [56] emphasised the importance of addressing emotional loneliness and psychological pain among older adults as key preventive measures. Cheng et al. [55] performed a bibliometric analysis of psychological pain research, noting exponential growth in studies since 2010, reflecting rising clinical recognition of psychological pain as an intervention target. Moreover, Morales & Barros [57] conducted a qualitative review identifying core psychological states and traits associated with suicidal psychological pain. Their findings describe transient states such as hopelessness, loneliness, and unbearable psychological suffering, as well as relatively stable traits including alexithymia, emotional dysregulation, and interpersonal difficulties. The authors emphasise that both domains—states and traits—are clinically relevant and modifiable through psychotherapeutic intervention, underscoring the centrality of psychological pain in suicide risk and the need for targeted interventions aimed at broadening emotional and behavioural coping repertoires. Overall, prior reviews [53,54,55,56,57] indicate the relevance of cognitive-behavioural and mindfulness-based strategies and identify psychological pain as both a state and trait phenomenon, supporting psychological pain as a meaningful intervention target.

### 3.5. Core Therapeutic Components

Across the reviewed studies, several recurring therapeutic components can be identified, although their specific contribution to reducing psychological pain within suicide prevention frameworks remains heterogeneous and is not consistently directly assessed. These components are derived from interventions with different primary targets and levels of evidence, and therefore should be interpreted as indicative rather than definitive.

1. Emotional Regulation and Acceptance—Teaching skills to identify, understand, and manage intense emotional experiences that constitute psychological pain [58], while helping individuals develop different relationships with psychological pain through acceptance-based approaches.
2. Cognitive Restructuring—Addressing distorted thinking patterns that amplify psychological pain, including catastrophic thinking, hopelessness, and negative self-evaluation [59].
3. Social Connectedness—Restoring a sense of belonging and interpersonal security.
4. Meaning Reconstruction—Enhancing purpose and coherence amid suffering.
5. Problem-Solving Skills—Enhancing ability to generate alternative solutions to problems contributing to psychological pain and suicidal thoughts.
6. Safety Planning and Relapse Prevention—Structured tools for crisis management.

### 3.6. Gaps and Priorities in Research on Psychological Pain and Suicide

Research on interventions targeting psychological pain in suicidal individuals presents several methodological challenges, including the use of heterogeneous measurement tools, limited standardisation, and uncertainty regarding optimal timing (e.g., acute vs. post-acute phases). In addition, the evidence base comprises a range of study designs (e.g., RCTs, non-randomised designs, and reviews), which differ in methodological robustness and scope. Consequently, the current synthesis should be interpreted as descriptive and exploratory. Mechanism-focused analyses—particularly those including pre- and post-intervention assessments of psychological pain—have been proposed as relevant for clarifying treatment effects. However, ethical and feasibility constraints inherent to high-risk populations may limit the implementation of such designs. Similarly, implementation-related factors, such as minimal-contact formats, follow-up procedures, and remote delivery, have been described in the literature as potential strategies to enhance safety and adherence, although their specific impact remains variable across studies. Some interventions, including group-based and digital approaches grounded in cognitive behavioural or acceptance-based frameworks, have been frequently described in the literature addressing suicidal populations, particularly in post-discharge contexts. Nevertheless, their specific effects on psychological pain are not consistently or directly assessed.

Despite the theoretical relevance attributed to psychological pain, relatively few interventions explicitly target this construct. Most approaches focus on related symptoms or behavioural outcomes, and only a limited number of studies evaluate psychological pain as a primary outcome or mediator. Overall, these findings indicate the need for further research aimed at improving conceptual clarity, measurement consistency, and the evaluation of psychological pain within intervention studies.

### 3.7. Therapeutic Proposal and Protocol

PSICOdolor: An Online Group Intervention Targeting Psychological Pain in Suicide Prevention.

In light of the findings identified in the scoping review, and considering the heterogeneity and limitations of the available evidence, a structured intervention protocol was developed as an exploratory and theory-informed proposal. Rather than representing a direct synthesis of the results, the protocol is intended to illustrate how different therapeutic components described in the literature may be integrated into a clinically applicable format. The proposed programme integrates cognitive-behavioural, emotion-regulation, and meaning-focused strategies highlighted in previous research, while placing psychological pain as the central therapeutic target. This approach differentiates the present protocol from most existing models, in which psychological pain is often addressed indirectly or as a secondary outcome, and only a limited number of interventions have explicitly targeted it as a primary and structured therapeutic focus.

#### 3.7.1. Design

The ‘PSICOdolor’ intervention was designed within the ‘SURVIVE 2 Project’, a multi-site-cohort study with nested randomised-controlled clinical trials. The main objective of the SURVIVE 2 is to expand on a previous cohort study (*N* = 3600) to investigate suicidal behaviour in Spain and to test the efficacy of secondary prevention strategies. Patients belonging to the SURVIVE 2 study cohort who had made a previous suicide attempt and were experiencing psychological pain will be invited to participate in the RCT comparing the Online Group Intervention Targeting Psychological Pain (PSICOdolor) + TAU vs. TAU.

#### 3.7.2. Participants

##### Eligibility Criteria

Inclusion criteria: (1) ≥18 years of age, (2) having attempted suicide within the previous 15 days, (3) presenting a significant score (≥31) on ‘psychological pain’ assessed by the Psychache Scale [60,61], (4) digital literacy (i.e., capacity to participate in an online intervention using a desktop computer or laptop), (5) agreement to participate in the study and sign the informed consent. Exclusion criteria: (1) lifetime diagnosis of schizophrenia, schizoaffective, bipolar, or psychotic disorder. The decision is driven by methodological, clinical, and safety considerations—rather than by reduced clinical relevance.

##### Calculation of Sampling Power

A total of 160 individuals from the SURVIVE 2 cohort will be included. Calculations with G*Power version 3.1 (for α = 0.05; 1 − β = 0.80; *d* = 0.5; difference between two independent groups) indicate that the sample should consist of 64 participants in each treatment arm, and thus a total of 128 participants. A sample of 80 participants (per treatment arm) is determined, considering a drop-out rate of 25%.

##### Recruitment

Participants will be recruited at emergency departments of public, general, and university hospitals in Catalonia (Hospital Clínic de Barcelona; Consorci Corporació Sanitària Parc Taulí; Hospital del Mar); Madrid (Hospital Clínico San Carlos; Hospital Universitario La Paz; Fundación Jiménez Díaz; Hospital Universitario Gregorio Marañón; Universidad Complutense de Madrid); Basque Country (Hospital Universitario Araba-Santiago); and Asturias (Hospital Universitario Central de Asturias–Universidad de Oviedo).

#### 3.7.3. Study Procedures

The participants will be assessed using a battery of clinical interviews and participant-reported outcomes at five time-points: baseline (V0—within 15 days of the suicide attempt), month 3 (V1—conducted remotely), month 6 (V2), month 9 (V3—conducted remotely), and month 12 (V4—last visit). In addition, participants will be assessed for the dimension of psychological pain before and after the intervention programme. All the assessments will be performed through an electronic, patient-reported outcome system (MeMind, version v1.0.23).

#### 3.7.4. Outcome Measures

The primary outcome will be subsequent suicide attempts (and/or suicide mortality) captured across a 12-month follow-up. The time elapsed between the index suicide attempt and the recurrence will also be considered. Psychological pain will be considered a mediating mechanism through which the intervention is expected to reduce suicide-related outcomes. The following variables will be collected: (1) sociodemographic data, (2) mental health diagnosis, (3) medical history and current treatments, (4) Psychache Scale [60,61], (5) depressive symptoms (Patient Health Questionnaire-9; PHQ-9) [62], (6) anxiety symptoms (Generalized Anxiety Disorder Scale; GAD-7) [63], (7) Health Questionnaire (EUROQOL-5D) [64], (8) Visual Analog Scale to measure Psychological and Physical Pain (PPP-VAS) [65,66], and the (9) Columbia Suicide Severity Rating Scale (C-SSRS) [67].

#### 3.7.5. Description of the Intervention According to TIDieR [68]

The psychological intervention based on CBT and ACT consists of a total of 10 sessions: an initial individual session and nine weekly group sessions, each lasting one h, delivered online. The content includes psychoeducation on psychological pain, depression, hopelessness, and negative internal emotional experiences; cognitive restructuring (CR) of maladaptive thoughts and unpleasant emotions; specific work on hopelessness and feelings of not belonging; adaptive coping strategies; training in emotional regulation skills; and relapse prevention [69,70,71,72,73].

The intervention is structured into progressive thematic modules (Table 2). The first sessions (Modules 0 and 1) introduce the treatment and explore the concept of psychological pain. Modules 2 to 4 then focus on emotional awareness, emotional self-regulation, self-esteem and communication skills. Modules 5 and 6 address awareness of thoughts, with special attention to cognitive biases, and learning more adaptive alternative thoughts. Subsequently, Modules 7 and 8 delve deeper into mental health awareness, coping, and crisis resolution. Finally, Module 9 is dedicated to relapse prevention and reflection on achievements. Throughout the programme, participants work on detecting warning signs, developing a safety plan, and promoting the therapeutic alliance, as well as being assigned homework tasks before and after each session. Intervention strategies and resources are administered by trained psychologists experienced in suicide risk assessment and crisis management.

##### Risk Management Procedures and Safety Considerations

Risk management and safety procedures will be implemented throughout the intervention to ensure timely detection and management of acute distress or elevated suicide risk. If, at any time during the intervention, the professional detects indicators of high suicide risk, severe decompensation of the underlying mental disorder, or non-adherence to mental health treatment, the participant will be advised to promptly schedule or bring forward an appointment with their referring psychiatrist or mental health professional. When clinically indicated, participants will be instructed to contact their referral health centre, emergency department, or 24 h emergency telephone services. In the event of acute distress during an online group session, the professional will prioritise participant safety, and if necessary, activate appropriate referral pathways. These procedures aim to ensure continuity of care and to minimise potential risks associated with remote group-based interventions.

#### 3.7.6. Control Condition

TAU is a heterogeneous combination of strategies which includes visits to specialised mental health services or pharmacotherapy. Routine procedures applied at each participating centre are considered TAU, including non-specific interventions for suicide prevention. All participants receive TAU regardless of their assignment to the specific intervention.

#### 3.7.7. Ethics

The study is conducted in accordance with the Declaration of Helsinki, and the protocol has been reviewed and approved by ethical committees at each participating site with respect to compliance with applicable research and human subjects’ regulations. Consent will be obtained independently for participation in the cohort study and optional procedures (i.e., RCTs).

#### 3.7.8. Dissemination Plans

Study results will be written for publication following completion of study data collection and data analyses.

#### 3.7.9. Protocol Version

V1.0 Dated 10 December 2025.

## 4. Discussion

The present scoping review underscores psychological pain as a central yet underutilised construct in suicide prevention, consistently identified across theoretical and empirical literature as a proximal mechanism driving suicidal ideation. Our results showed that existing interventions—such as CBT, ACT, MBCT, brief contact approaches, and interpersonal therapies—may reduce psychological pain indirectly through improvements in emotion regulation, cognitive flexibility, and relational connectedness, but few treatments target psychological pain explicitly [33]. Most of these interventions focused on other outcomes, such as suicidal ideation, and used psychological distress as a secondary outcome. However, the number of studies or unpublished trials about psychological interventions targeting psychological pain is scarce. Similarly, there are few pharmacological interventions that specifically target psychological pain as an outcome. Indeed, several reviews highlight the importance of measuring psychological pain as an outcome, especially in suicide.

Only one intervention [43] specifically targeted psychological pain using a CBT approach. The Psychological Pain Theory-Based Cognitive Therapy based its intervention on the three-dimensional model of psychological pain, including pain arousal, painful feelings, and pain avoidance. Zou et al. [43] created a 16-session intervention based in CBT, with several components such as psychoeducation, socratic questioning of irrational beliefs, cognitive restructuring, and problem-solving skills. This pilot study showed a small effect size in reducing psychological pain. Other studies using third-generation psychotherapies [38,39,40] showed the reduction in psychological pain with a large effect size, although not targeting it specifically. Psychological pain includes emotional distress and self-brokenness components in its definition. In this sense, third-generation approaches focus more on emotions and self than CBT, which focuses more on cognition, explaining, then, the differences in effect sizes. An additional explanation for the smaller effect size observed in the CBT-based pilot study [43], compared with some third-generation interventions, may lie in differences in therapeutic mechanisms and clinical context. Although the intervention explicitly included components aimed at reducing psychological pain, it was conceived as a multi-component post-suicide attempt programme addressing several risk factors simultaneously, rather than as a treatment exclusively centred on psychache. Third-generation therapies, in contrast, may exert a stronger impact on psychological pain through processes such as acceptance, mindfulness, and reduction in experiential avoidance, which are conceptually close to the experience of unbearable mental pain. In addition, the acute clinical profile of the sample, the post-discharge context, and the brief online group format may have reduced the magnitude of the observed effects.

Pharmacological approaches, particularly rapid-acting agents (i.e., ketamine), have attracted growing attention due to their capacity to produce short-term reductions in depressive symptoms, suicidal ideation, and affective distress [75,76,77]. While these agents may offer important symptomatic relief and create a window of opportunity for engagement in care, their use in isolation raises several conceptual and clinical limitations. Concerns have been raised regarding feasibility, repeated administration, and the lack of clarity surrounding long-term outcomes when rapid-acting agents are used without concurrent psychotherapeutic support [78]. Moreover, pharmacological interventions alone do not directly address the cognitive, emotional, interpersonal, and existential processes that contribute to the persistence and recurrence of psychological pain. While psychological interventions work primarily through cognitive, emotional, and relational mechanisms—fostering tolerance, coherence, and connectedness, pharmacological interventions may provide symptomatic relief of psychological pain, enabling engagement in psychotherapy. The evidence suggests that the future may lie in integrative models, combining fast-acting agents (e.g., ketamine, buprenorphine) with structured psychotherapeutic work on meaning and tolerability [79]. In this context, the PSICOdolor protocol incorporates therapeutic components aimed at enhancing emotional awareness, emotional regulation, and cognitive processing, which directly target the experience of psychological pain. Therefore, implementing after rapid-acting pharmacological treatment, such as ketamine, following a suicidal crisis may help to sustain the short-term therapeutic effects of these agents. While pharmacological interventions may provide rapid relief of affective distress and suicidal ideation, the psychotherapeutic components of PSICOdolor protocol are designed to address the underlying mechanisms involved in the development and persistence of psychological pain, which could contribute to longer-term stabilisation and relapse prevention.

Furthermore, reducing psychological pain may be equivalent to reducing suicidal ideation, since psychological pain is at the foundation of suicidal ideation [11]. The reviewed studies show that both psychological pain and suicidal ideation were reduced after intervention [38,39,40,43,50,52]. Indeed, the reduction in psychological pain may prevent suicide attempt relapses [42], showing its relation not only with suicidal ideation but also with suicide behaviour. Finally, psychological pain is a transdiagnostic marker of psychopathology [1,2]; thus, targeting psychological pain may induce improvements in other psychopathologies such as depression, anxiety or hopelessness.

Thus, the evidence reviewed suggests the need for interventions that move beyond pharmacological approaches and symptom-oriented or behavioural strategies by addressing the specific mechanisms underlying psychological pain, including overwhelming affective distress, maladaptive cognitions (e.g., perceived burdensomeness), experiential avoidance, and disconnection from self, others, and meaning. In response to these gaps, the ‘PSICOdolor’ protocol integrates CBT and ACT principles in an online group format specifically designed to reduce psychological pain following a suicide attempt, combining emotional regulation, cognitive restructuring, interpersonal and meaning-oriented strategies, and safety planning. Beyond the theoretical relevance of psychological pain as a core mechanism underlying suicidal behaviour, the implementation of structured intervention protocols specifically targeting this construct may have important clinical implications. The proposed protocol translates the available evidence into a manualised and replicable intervention that can be integrated into routine care after a suicide attempt, a period characterised by high vulnerability and elevated risk of relapse [80]. The group-based and online format may facilitate accessibility, continuity of care after discharge, and adherence to treatment, which are common challenges in suicide prevention programmes [81]. From a clinical perspective, the use of structured protocols may also contribute to greater standardisation of care, allowing clinicians to address psychological pain in a systematic way rather than as a secondary or implicit treatment target. If supported by the results of the ongoing RCT, the proposed intervention could represent a feasible and scalable strategy to complement treatment as usual, contributing to more precise and mechanism-focused approaches in suicide prevention. In this sense, integrating protocols specifically designed to reduce psychological pain into clinical practice may help to improve recovery trajectories and reduce the likelihood of repeated suicide attempts.

Although this scoping review integrates diverse psychological, pharmacological, and theoretical evidence to inform a structured, manualised intervention, several key limitations should be highlighted. First, due to the heterogeneity of the available studies in terms of design, populations, interventions, and outcomes, conducting a systematic review was not feasible. Second, the review was not pre-registered, which may limit methodological transparency and increase the potential for bias. Given the exploratory nature of the review and the diversity of study designs, no formal risk-of-bias tool was applied; however, methodological characteristics of the included studies were considered during the interpretation of findings. The absence of a formal critical appraisal limits the ability to draw conclusions regarding the relative strength of the evidence. Finally, the inclusion of studies with very different methodologies—while acceptable within a scoping or narrative approach—should be acknowledged as a limitation that may affect the generalisability and strength of the conclusions drawn. Of note, the body of evidence included in the review comprises heterogeneous sources (e.g., RCTs, non-randomised designs, and reviews), with substantial variability in methodological robustness. Accordingly, the synthesis should be interpreted as exploratory and descriptive.

An additional aspect that should be considered is the requirement of a minimum level of digital literacy, as the proposed intervention is designed to be delivered in an online group format. Although this modality offers advantages in terms of accessibility and continuity of care after discharge, it may represent a barrier for some patients, particularly older adults, who may have less familiarity with digital technologies. This is especially relevant given that psychological pain is also highly prevalent in older populations. Adaptations to the protocol will be considered wherever possible, such as the inclusion of additional technical support or simplified digital procedures, in order to ensure accessibility for people with lower digital literacy and to improve the generalisability of the intervention.

Future research should systematically examine psychological pain as an outcome and mediator in clinical trials, compare interventions explicitly focused on psychological pain with broader symptom-based approaches, and clarify optimal intervention timing, especially in digital or remote formats that may enhance post-discharge continuity of care. Understanding that psychological pain represents a state or trait characteristic (or both) may help researchers to delineate a place for psychological pain in suicide prediction. Whether psychological pain plays a role in short-term prediction of suicidal ideation and suicide events is a topic for future ecological momentary assessment (EMA) studies [3,82]. In addition, future studies may explore the potential benefits of maintenance sessions delivered after the core intervention as a strategy to maintain reductions in psychological pain and consolidate therapeutic gains over longer follow-up periods. The proposed intervention protocol, while theory-driven and evidence-informed, awaits empirical validation through the nested RCT. Overall, targeting psychological pain directly may strengthen suicide prevention efforts, enhancing recovery trajectories and reducing relapse risk following a suicide attempt.

### Adaptation for Low-Technology Contexts

While the proposed intervention is designed as an online group programme, considerations regarding accessibility are particularly relevant for populations with limited digital literacy, including older adults. Psychological pain has been described as prevalent across age groups, and barriers to technology use may restrict access to fully digital formats. In this context, the therapeutic components outlined in the protocol may be adapted to low-tech delivery modalities. For example, synchronous group sessions could be supported by simplified digital platforms requiring minimal technical skills. Key elements such as psychoeducation, cognitive restructuring, and emotional regulation strategies may also be delivered through printed materials. Hybrid approaches combining remote follow-up contacts by telephone may further enhance accessibility and continuity of care. In addition, involving caregivers or support networks, when appropriate, may facilitate engagement and adherence in individuals with limited familiarity with digital tools. Future studies should examine how varying delivery formats affect participant engagement, the therapeutic alliance, and outcomes associated with psychological pain.

## 5. Conclusions

Psychological pain represents a core mechanism underlying suicidal behaviour and a clinically meaningful target for intervention, yet it remains under-assessed and insufficiently addressed in routine care. The present scoping review identifies key therapeutic components targeting psychological pain and synthesises them into a structured, online, psychological-based group intervention tailored for individuals after a suicide attempt. The ‘PSICOdolor’ protocol responds to critical gaps by directly addressing psychological pain within a multi-site RCT design. Integrating psychological pain into standard assessment and treatment frameworks may enhance the precision and effectiveness of suicide prevention strategies.

## Figures and Tables

**Table 1:** Narrative synthesis of evidence on psychological pain and suicide.

Section	Focus of the Section	Type of Evidence Synthesised	Main Contribution
Psychological interventions	Psychological interventions addressing psychological pain explicitly or indirectly	RCT [38,39,40,41]; quasi-experimental design [42]; pilot and feasibility study [43]; case report [44]; and non-experimental studies [45,46,47,48,49]	Demonstrates that psychotherapeutic approaches can reduce psychological pain and suicide-related outcomes through emotion regulation, cognitive restructuring, interpersonal connectedness, acceptance-based strategies, and meaning-oriented processes, while highlighting heterogeneity in intervention focus and outcome assessment
Pharmacological interventions	Rapid-acting pharmacological strategies targeting affective distress and psychological pain	Experimental research [50]; and narrative reviews [51,52]	Certain pharmacological agents may rapidly reduce psychological pain and suicidal ideation in acute phases, while underscoring limitations related to sustainability of effects, feasibility, and the need to integrate pharmacological approaches with psychotherapeutic interventions
Ongoing and unpublished trials	Emerging psychological and experimental interventions targeting suicide risk and psychological pain	Ongoing and recently completed clinical trials and study protocols	Provides insight into current research directions, including trait-level vulnerability to psychological pain, comparative efficacy of brief psychotherapies (ASSIP vs. ACT), and digitally supported group interventions, highlighting innovation in mechanisms assessment
Literature reviews	Synthesis of existing evidence on suicide prevention and psychological pain	Systematic review [53]; narrative review [54]; bibliometric [55]; and qualitative analyses [56,57]	Consolidates evidence supporting psychological pain as a clinically relevant and modifiable target in suicide prevention, identifies intervention modalities, and emphasises the importance of personalised mechanism-focused approaches

Note. RCT: randomised controlled trial; ASSIP: Attempted Suicide Short Intervention Program; ACT: Acceptance and Commitment Therapy.

**Table 2:** Structure of the intervention for psychological pain in suicide prevention.

No.	Component	Themes	Content
0	Individual session	Introduction to treatment. Concept of psychological pain (I)	Presentation of treatment rationale and programme overview. Group rules. Calendar. Explanation of online platform. Establishment of safety plan. Homework: Review safety plan; bring doubts/corrections next session.
1	Group session	Introduction to treatment. Concept of psychological pain (II)	Participant introductions. Homework review. Psychoeducation: nature of emotions. Definition of psychological pain. Emotional regulation: diaphragmatic breathing. Homework: practice diaphragmatic breathing.
2	Group session	Awareness of feelings. Emotional regulation (I)	Homework review. Concept and importance of emotional regulation. Emotional regulation skills: changing body chemistry to reduce intense emotions. Relaxation strategies. Homework: practice progressive muscle relaxation.
3	Group session	Awareness of feelings. Emotional regulation (II)	Homework review. Psychological pain and disconnection from self, future, and world. Emotional regulation skills: awareness and anchoring in the present. Mindfulness exercises. Homework: practice mindfulness and emotional awareness.
4	Group session	Awareness of feelings. Self-esteem and communication strategies	Homework review. Psychoeducation on self-concept and self-esteem. Psychoeducation on communication styles. Training in assertive communication. Homework: practice assertive communication.
5	Group session	Awareness of thoughts (I). Alternative thoughts	Homework review. Introduction to ABC model. Cognitive restructuring of maladaptive thoughts. Homework: thought record.
6	Group session	Awareness of thoughts (II). Cognitive biases	Homework review. Identification of cognitive biases. Cognitive flexibility training. Questioning validity and usefulness of thoughts. Generating adaptive alternatives. Homework: identify distortions and practice cognitive strategies.
7	Group session	Awareness of mental health (I)	Homework review. Psychoeducation on mental health in the context of unpleasant emotional experiences and distress (depression, hopelessness, and thwarted belongingness) [74]. Behavioural activation. Homework: design behavioural activation plan.
8	Group session	Awareness of mental health (II)	Homework review. Crisis management and resolution. Problem-solving training. Homework: practice problem-solving and emotional regulation plan.
9	Group session	Recognising achievements and looking to the future. Relapse prevention	Homework review. Recapitulation of skills. Evaluation of progress. Discussion of benefits. Anticipating difficulties. Final closure and encouragement to continue practice.

Note. Transversal elements include recognition of warning signs and development of a safety plan, promotion of therapeutic alliance, and structured homework tasks before and after each session.

## Data Availability

Publicly archived datasets that were analysed or generated during the study will be included.

## Supplementary Materials

The following supporting information can be downloaded at https://www.mdpi.com/article/10.3390/jcm15083124/s1. Supplementary File S1. Preferred Reporting Items for Systematic reviews and Meta-Analyses extension for Scoping Reviews (PRISMA-ScR) Checklist; Supplementary File S2. Search strategy.

## Abbreviations

The following abbreviations are used in this manuscript:

CBT	Cognitive Behavioural Therapy
DBT	Dialectical Behaviour Therapy
ACT	Acceptance and Commitment Therapy
MBCT	Mindfulness-Based Cognitive Therapy
PRISMA-ScR	Preferred Reporting Items for Systematic reviews and Meta-Analyses extension for Scoping Reviews
RCT	Randomised Controlled Trial
CBT-SP	Cognitive Behavioural Therapy for Suicide Prevention
ASSIP	Attempted Suicide Short Intervention Program
TAU	Treatment As Usual
CR	Cognitive Restructuring
PHQ-9	Patient Health Questionnaire-9
GAD-7	Generalized Anxiety Disorder Scale
EUROQOL-5D	Health Questionnaire
PPP-VAS	Visual Analog Scale to Measure Psychological and Physical Pain
C-SSRS	Columbia Suicide Severity Rating Scale
EMA	Ecological Momentary Assessment
